# Can antimicrobial blue light contribute to resistance development? Genome-wide analysis revealed aBL-protective genes in *Escherichia coli*

**DOI:** 10.1128/spectrum.02490-23

**Published:** 2023-12-08

**Authors:** Beata Kruszewska-Naczk, Mariusz Grinholc, Krzysztof Waleron, Julia Elisabeth Bandow, Aleksandra Rapacka-Zdończyk

**Affiliations:** 1 Laboratory of Photobiology and Molecular Diagnostics, Intercollegiate Faculty of Biotechnology, University of Gdansk and Medical University of Gdansk, Gdansk, Poland; 2 Department of Pharmaceutical Microbiology, Faculty of Pharmacy, Medical University of Gdansk, Gdansk, Poland; 3 Applied Microbiology, Faculty of Biology and Biotechnology, Ruhr University Bochum, Universitätsstraße, Bochum, Germany; Emory University, Atlanta, Georgia, USA

**Keywords:** antimicrobial blue light, *Escherichia coli*, genome-wide analysis, hypersensitive mutants, resistance

## Abstract

Antimicrobial blue light (aBL) is a promising non-antibiotic approach to fighting multidrug-resistant bacteria. However, the complete mechanism of aBL action is not fully understood yet. This study contributes to a better understanding that the response to aBL depends on many factors and that it is hardly possible to identify a predominant mechanism underlying microbial sensitivity to photoinactivation. The results of this study provide insights into genetic changes that may lead to bacterial survival at higher aBL doses, giving rise to aBL-resistant strains. To our best knowledge, this is the first study concerning genome-wide mutant testing of aBL. We managed to identify 64 single-gene mutants that lacked certain protective genes expressing aBL-increased sensitivity.

**IMPORTANCE:**

Increasing antibiotic resistance and the lack of new antibiotic-like compounds to combat bacterial resistance are significant problems of modern medicine. The development of new alternative therapeutic strategies is extremely important. Antimicrobial blue light (aBL) is an innovative approach to combat multidrug-resistant microorganisms. aBL has a multitarget mode of action; however, the full mechanism of aBL antibacterial action requires further investigation. In addition, the potential risk of resistance development to this treatment should be considered.

## INTRODUCTION

According to data from the World Health Organization (1), an estimated 600 million cases of foodborne diseases and 420,000 deaths related to pathogens occur worldwide each year. Food products, especially minimally processed foods such as raw seafood, fresh vegetables, fruits, and raw juices, as well as food processing environments, are vulnerable to diarrheal disease pathogens. Consumer awareness of the harmfulness of preservatives and pesticides, as well as the potential loss of foods’ nutritional and organoleptic values, increases the need for the development of sterilization technology based on non-thermal approaches (2).

*Escherichia coli* (*E. coli*) is an intensively studied bacterial species widely present in the environment. Usually considered a harmless commensal bacterium, pathogenic strains are capable of causing diseases in humans and animals (3). An example of such pathogenic strains is the verotoxigenic foodborne pathogen *E. coli* O157:H7, which causes hemorrhagic colitis, hemolytic uremic syndrome, or even death (4). Multiple studies worldwide document increasing antibiotic resistance and associated healthcare threats (5 – 10). *E. coli* O157:H7 is the third leading cause of life-threatening foodborne illnesses among pathogens, next to *Salmonella* and *Listeria monocytogenes* (11). In the USA alone, the *E. coli* O157:H7 serotype causes 73,000 illnesses annually (12). The lack of new antibiotic-like compounds to combat bacterial resistance is a significant concern of modern medicine. According to *The Review on Antimicrobial Resistance*, it is estimated that by 2050, if new solutions do not slow down the rise in the drug resistance of pathogens, 10 million lives a year will be at risk due to multidrug-resistant infections. Without new effective antimicrobials, key medical procedures could become too dangerous to perform (13). Thus, the development of new alternative therapeutic strategies for treating multidrug-resistant infections is extremely crucial.

Antimicrobial blue light (aBL), an innovative light-based non-antibiotic and non-thermal approach to fighting multidrug-resistant microbes, has attracted increasing attention lately. It is believed to have a multitarget mode of action caused by the rapid reaction of reactive oxygen species (ROS) with a wide range of cellular macromolecules such as proteins, lipids, and nucleic acids (DNA and RNA), which results in cell death (14, 15). According to Hyun and Lee, aBL has antimicrobial potential against foodborne pathogens, is “eco-friendly,” and is a cheap and safe alternative to thermal approaches and ultraviolet (UV) radiation (16). Nevertheless, the complete mechanism of the antibacterial action of aBL is not fully understood yet and needs further analysis (17, 18). Moreover, the potential risk of resistance development should also be considered. In previous reports, our team has shown that the development of tolerance to aBL is possible for both gram-positive (19, 20) and gram-negative (21) bacteria. Nevertheless, no aBL resistance has been demonstrated so far.

The main aim of this study was to identify genes and proteins that are crucial for the bacterial response to aBL in *E. coli*. The resource for genome-wide testing of mutational effects was the Keio knockout collection, a set of precisely defined, single-gene deletions of non-essential genes in the strain background of *E. coli* BW25113 (22). The entire *E. coli* Keio collection was screened to distinguish single-gene mutants that express the aBL-hypersensitive phenotype.

## MATERIALS AND METHODS

**Fig 1:**
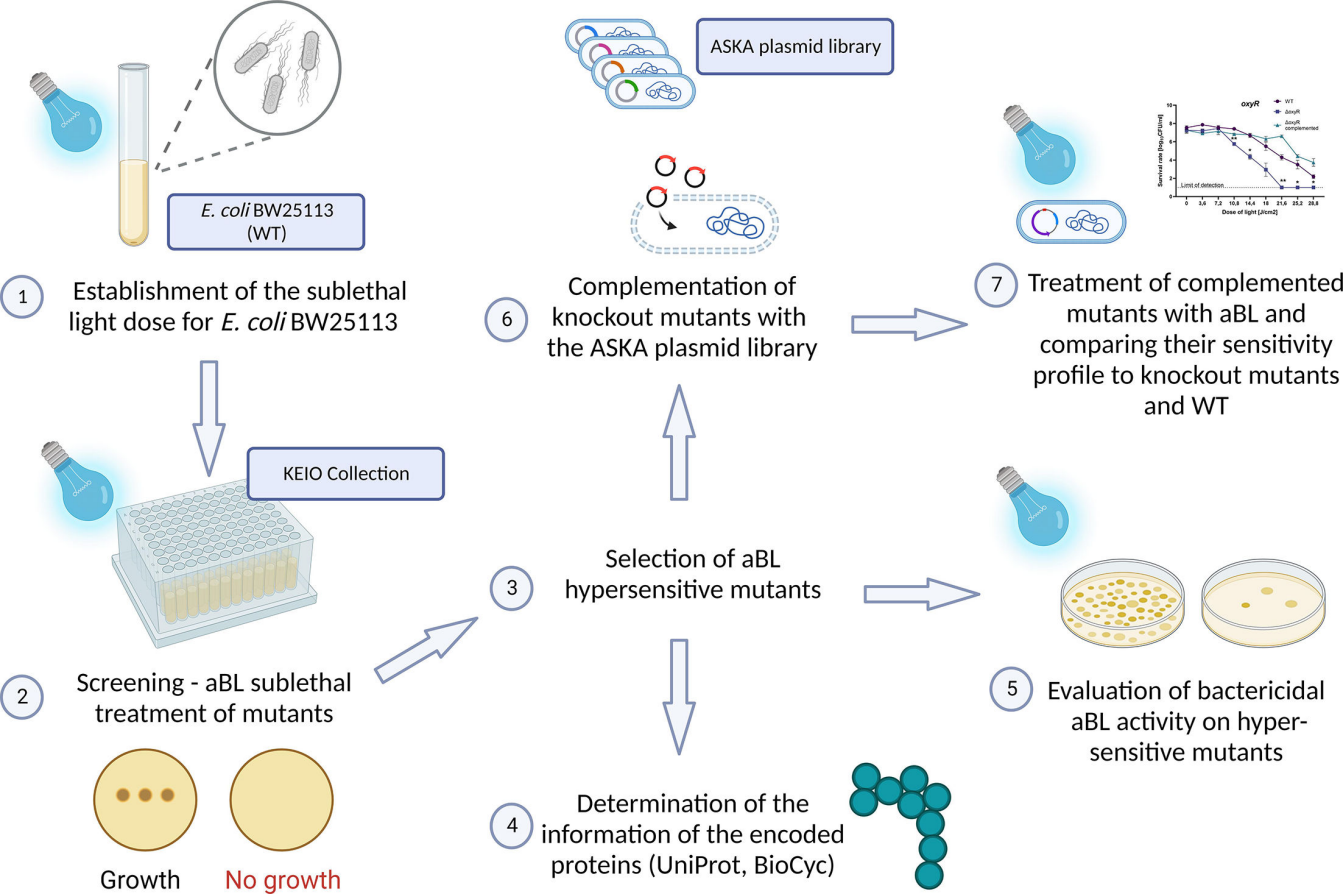
Experimental workflow.

The experimental workflow is presented in Fig. 1.

### Strains and culture conditions

Analyses were performed using a collection of *E. coli* BW25113 single-gene knockout mutants [the Keio collection, 3,985 strains (22)] and a complete set of ORF clones of *E. coli* (ASKA plasmid library) kindly provided by the National BioResource Project and the National Institute of Genetics [Shizuoka, Japan (23)].

All the strains were cultured in Luria–Bertani (LB) broth (BTL, Łódź, Poland) or LB agar medium (A&A, Gdansk, Poland) at 37°C under aerobic conditions in an orbital incubator (Innova 40, Brunswick, Germany) at 150 rpm. Knockout mutants were maintained in the presence of 15 µg/mL of kanamycin, whereas strains harboring plasmids were treated in the presence of 150 µg/mL chloramphenicol. The strains were stored in 96-deep well plates filled with LB, and 15% glycerol medium was administered to them for storage at −80°C. Before the cells were used, they were freshly stamped into new microtiter plates filled with the LB medium and incubated overnight (16–20 h) in an orbital incubator at 150 rpm.

### Chemicals

Kanamycin sulfate was purchased from Gibco (Paisley, UK), and chloramphenicol, from Sigma-Aldrich (Darmstadt, Germany). Stock solutions, at concentrations of 15 or 150 mg/mL, respectively, were prepared in ddH_2_O and stored at −20°C. Isopropyl β-d-1-thiogalactopyranoside (IPTG) (100 mg/mL; Blirt, Gdansk, Poland) stock solution was prepared and stored at −20°C.

### Light source

Irradiation was performed with an LED light source that emitted blue light (λmax 415 nm, irradiance 25 mW/cm^2^; Cezos, Gdynia, Poland). The light source allows 96 samples to be exposed to irradiation simultaneously. The LED light source was constructed to reach a homogeneous light distribution, and it is equipped with an optical lens to allow the equal distribution of light to ensure that equal doses of light are able to reach each sample. The emission spectra of light sources were measured using a Digikrom CM110 spectrograph (CVI Laser Corporation, USA) equipped with a ST-6V CCD camera (SBIG, USA). Irradiance was measured for each illumination spot (referring to each of the 96 wells) to evidence that the light distribution is homogenous and the differences between light densities do not exceed 10% (except for a few fields, which were taken into account when performing the experiments). To increase power homogeneity, the 96-well plates were placed in different orientations relative to the light source. The picture of the LED light source and the map of irradiance distribution over the illuminated area were presented in Fig. S1.

### aBL sensitivity of the *E. coli* parental strain BW25113

To quantify the aBL sensitivity of the *E. coli* BW25113 parental strain, microbial overnight cultures were adjusted to an optical density of 0.5 McFarland (McF; 5 × 10^7^ CFU/mL). Next, aliquots of 100 µL per well were transferred to 96-well microtiter plates and irradiated with different light doses (0–64.8 J/cm^2^). After their illumination, 10 µL aliquots of each sample were serially diluted 10 times in phosphate-buffered saline (PBS, Sigma-Aldrich, Germany) to generate dilutions of 10^−1^–10^−4^, which were streaked horizontally on plates. The plates were incubated at 37°C for 16–20 h, after which colonies were counted to estimate the survival rate, expressed in log_10_ CFU/mL. Each experiment was performed thrice. The minimal duration of the killing of 99% of the cells (MDK_99_, defined further as the sublethal dose) of irradiation was estimated for application in a screening of the Keio collection.

### Screening of the Keio collection

The main focus of this experiment was to identify *E. coli* single-gene mutants, which are especially sensitive to aBL treatment. Overnight cultures of knockout mutants prepared in microtiter plates were diluted 10 times in PBS. From each well that contained mutant cultures, 2 µL was dropped onto an LB agar plate supplemented with kanamycin. After 1-h incubation at 37°C, the spots were subjected to aBL at the light dose assigned as sublethal for the parental *E. coli* BW25113 strain. Dark controls (without irradiation) were also prepared. Afterward, the plates were incubated at 37°C overnight. The experiment was performed in three biologically independent repetitions. For each mutant, the number of experiments in which no cells were observed after overnight incubation (post-irradiation) was summed up to give a score. Mutants with a score of 2 or 3 were classified as aBL-sensitive, as Krewing et al. (24) described for other stressors.

### aBL sensitivity of the selected mutants

This analysis was performed to obtain quantified profiles of the aBL sensitivity of mutants and to compare them to the parental strain profile. Microbial overnight cultures were adjusted to an optical density of 0.5 McF (5 × 10^7^ CFU/mL), and aliquots of 100 µL per well were transferred to 96-well microtiter plates and irradiated with different light doses (0–43.2 J/cm^2^). After the illumination, 10 µL aliquots of each sample were serially diluted 10 times in PBS to generate dilutions of 10^−1^–10^−4^, which were streaked horizontally on LB agar plates. The plates were incubated at 37°C for 16–20 h, and then, colonies were counted to estimate the survival rates. Each experiment was performed thrice for each mutant.

### Complementation

The experiment was conducted to check if the complementation of the deleted genes would restore the wild-type (WT) phenotype of aBL sensitivity to hypersensitive mutants. The selected mutants of the Keio collection were transformed with the plasmid pCA24N of the ASKA collection, which harbors the respective gene under the control of an IPTG-inducible promoter for complementation of the knockout and with the selective gene of chloramphenicol resistance (22). Plasmids of interest were isolated with the EndoFree Plasmid Maxi Kit (Syngen, Wrocław, Poland). Next, complementation was performed with a standard protocol using CaCl_2_ (Sigma-Aldrich, Germany). An overnight culture of a strain of interest was diluted at the ratio of 1:100 in a fresh LB medium with kanamycin. Then, the strains were incubated at 37°C in an orbital incubator (Innova 40, Brunswick, Germany) at 150 rpm to reach an optical density of 0.4–0.6. The bacterial suspension was centrifuged and suspended in a 50 mM CaCl_2_ cold solution in half volume of the primary culture and incubated on ice for 0.5 h. Next, the suspensions were centrifuged and suspended in 1 mL of the CaCl_2_ solution and incubated for 1 h. After this, 200 µL of the suspensions was transferred into new tubes, and approximately 200 ng of isolated plasmid DNA was added. No DNA was added to the control probe. Next, the probes were incubated for 1 h, transferred to 43°C for 3 min, and then immediately placed on ice for 3 min. Next, 1 mL of the LB medium was added, and the probes were incubated in the orbital rotator for 1 h at 37°C. After that, the probes were streaked on LB agar plates with kanamycin (growth control) or chloramphenicol to verify proper transformation. Additionally, the presence or absence of a gene of interest was verified by PCR reaction using specified primers dedicated to the ASKA collection (oligonucleotides were synthesized by Oligo, Warsaw, Poland). The complemented and verified strains were used in the next experiments.

### Evaluation of the optimized experimental conditions adequate for proper analysis of the *E. coli* BW25113 Δ*oxyR* deletion mutant and the complemented strain response to aBL

The experimental conditions were optimized with the use of one representative of the *E. coli* mutants (i.e., *E. coli* BW25113 Δ*oxyR* and its complemented derivative).

#### Evaluation of the IPTG concentration

Overnight cultures of the complemented Δ*oxyR* strain were diluted at the volume-to-volume (vol/vol) ratio of 1:100 in the fresh LB medium supplemented with specified antibiotics. The IPTG solution was added to the cultures to obtain total concentrations of 2, 1, and 0.5 µM. After 2 h of incubation (37°C, 150 rpm), the cultures were adjusted to 0.5 McF and treated with a range of light doses: 0–36 J/cm^2^. After the irradiation, aliquots were plated to determine CFU/mL, as described in previous experiments.

#### Evaluation of the IPTG induction time for the complemented mutant and choice of the bacterial growth phase for the deletion mutant

Two overnight cultures of the complemented Δ*oxyR* strain with specific antibiotics were prepared. IPTG was administered to the first culture at the start of the culture to obtain 1 µM of IPTG. Then, the samples were incubated at 37°C with shaking (at 150 rpm) for 16 h. The second overnight culture was diluted after 16 h of incubation (37°C, 150 rpm) at the vol/vol ratio of 1:100 in the fresh LB medium supplemented with specified antibiotics and 1 µM IPTG. After 2 h of incubation (37°C, 150 rpm), the cultures were diluted to 0.5 McF and then treated with 0–28.8 J/cm^2^ of light doses. After the irradiation, the aliquots were plated to determine the CFU/mL, as described in previous experiments. The culture time selection for knockout mutants was set up with the same experimental workflow but without the addition of IPTG. Each experiment was performed three times for each strain.

#### Evaluation of the impact of different IPTG concentrations on bacterial growth

The growth curve of complemented strains in a range of different IPTG concentrations was determined. Overnight cultures of the strain were diluted at the vol/vol ratio of 1:20 and supplemented with IPTG to obtain the final concentrations of 0–1,000 µM. The growth was monitored for 6 h in an EnVision Multilabel Plate Reader (PerkinElmer, USA). The OD_600_ value was measured every 15 min with incubation at 37°C with shaking (150 rpm). All the experiments were performed in three biological repetitions.

### aBL sensitivity of the knockout and complemented mutants

This experiment was designed to compare the aBL sensitivity profiles of the wild-type and knockout mutants and the complemented strains. Overnight cultures of the selected mutants were diluted at the vol/vol ratio of 1:100 in the fresh LB medium supplemented with specified antibiotics. The IPTG solution was added to the complemented mutant culture to obtain a total concentration of 1 µM. Then, the samples were incubated for 2 h at 37°C and 150 rpm. Next, the cultures were adjusted to a 0.5-McF optical density, and 100 µL aliquots were placed in 96-well plates. The previously prepared IPTG stock was added to the complemented mutant solutions to reach a 1 µM concentration. Then, the probes were irradiated with 0–28.8 J/cm^2^ doses of light. After the illumination, aliquots of each sample were serially diluted, streaked, and cultured as described previously. All the analyses were also performed for the parental strain of *E. coli*. All the experiments were performed thrice for each strain.

### Bioinformatical and statistical analysis

All the statistical analyses and figures were created using GraphPad Prism version 9.0 (GraphPad Software, Inc., CA, USA). The statistical differences between the groups were calculated using two-way analysis of variance (ANOVA) with *P* < 0.05 and Tukey’s multiple comparison tests. Functional analysis of hypersensitive genes was performed using the BioCyc database and the Omics Dashboard. Protein–protein functional interaction networks were analyzed using the STRING database with a medium score of confidence of 0.4. The analysis was performed using the data from the curated databases that were experimentally determined (25). The graphic figures were prepared with the use of BioRender.com (accessed on 13 November 2022).

## RESULTS

### Determination of the aBL sensitivity of the *E. coli* BW25113

**Fig 2:**
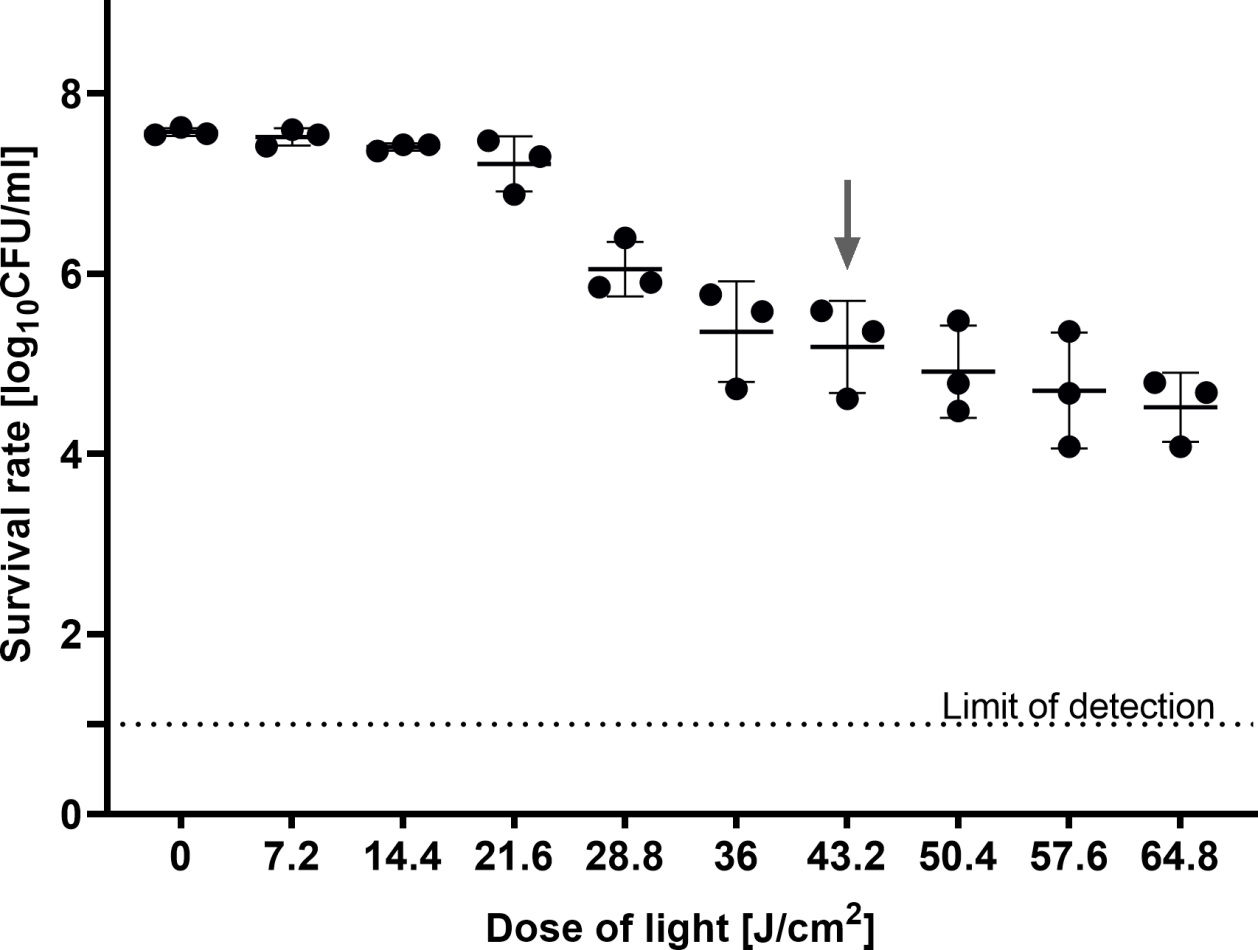
aBL sensitivity of the *E. coli* BW25113. The detection limit was 10 CFU/mL. The arrow indicates the sublethal treatment chosen for the screening of the Keio collection. Overnight cultures of the **BW25113** strain were diluted to 0.5 McF and irradiated with 0–64.8 J/cm^2^ light doses. The CFU/mL was estimated with serial dilutions of 10 µL aliquots of the irradiated samples and plated on LB agar. The plots present the reduction of log_10_ units of CFU/mL. The experiment was performed in a biological triplicate.

The first step was to determine the irradiation conditions that were sublethal (MDK_99_) for the wild-type *E. coli* BW25113 strain but could be lethal (≥MDK_99.9_) for aBL-sensitive mutants. The conditions that resulted in a 2 log_10_ reduction in treated parental strains for the Keio collection screening were determined as 43.2 J/cm^2^ (Fig. 2).

### Establishment of aBL-hypersensitive mutants

**Fig 3:**
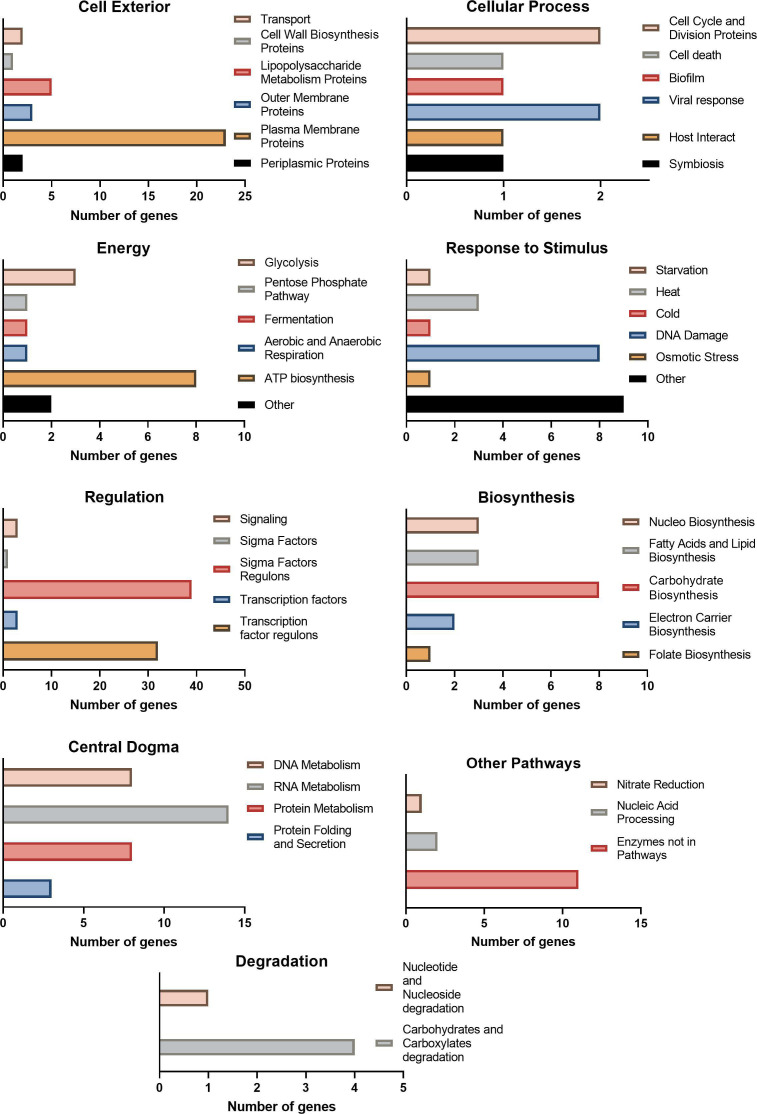
Functional analysis of genes important for surviving aBL with the BioCyc Database.

All the single-gene deletion mutants were treated with aBL at the light dose assigned as sublethal for the parental strain (43.2 J/cm^2^) in three biological repetitions. Screening analysis enabled the detection of 64 knockout mutants with increased sensitivity to aBL, which are listed in Table 1. The genes and their functions are compared in Table S1 (Supplement Materials) and Fig. 3. Some of the genes could be assigned different functional categories.

**TABLE 1:** List of 64 *E. coli* BW25113 single-gene mutants identified as “aBL-hypersensitive” (no growth after the aBL dose of 43.2 J/cm^2^ in at least two of three biological repetitions)^*a*^

Lp.	Gene name	Synonyms	Description	Number of points
**1**.	*atpA*	*papA // uncA*	ATP synthase subunit alpha	2
**2**.	*atpB*	*papD // uncB*	ATP synthase subunit a	3
**3**.	*atpC*	*papG // uncC*	ATP synthase epsilon chain	2
**4**.	*atpD*	*papB // uncD*	ATP synthase subunit beta	3
**5**.	*atpE*	*papH // uncE*	ATP synthase subunit c	3
**6**.	*atpF*	*papF // uncF*	ATP synthase subunit b	3
**7**.	*atpG*	*papC // uncG*	ATP synthase gamma chain	2
**8**.	*atpH*	*papE // uncH*	ATP synthase subunit delta	3
**9**.	*cpxA*	*rssE // ecfB // eup // ssd // ecf*	Sensor histidine kinase CpxA	2
**10**.	*cydD*	*htrD*	ATP-binding/permease protein CydD	2
**11**.	*dacA*	*Pfv*	D-Alanyl-D-alanine carboxypeptidase DacA	2
**12**.	*deoB*	*tlr // drm // thyR*	Phosphopentomutase	2
**13**.	*dnaJ*	*groP // grpC*	Chaperone protein DnaJ	2
**14**.	*dnaK*	*groPF // groPC // seg // grpF // grpC // groPAB*	Chaperone protein DnaK	3
**15**.	*ecnB*	*yjeU*	Entericidin B	2
**16**.	*fabH*		3-Oxoacyl-[acyl-carrier-protein] synthase 3	2
**17**.	*fimB*	*Pil*	Type 1 fimbriae regulatory protein FimB	2
**18**.	*gmhB*	*yaeD*	D-Glycero-beta-D-manno-heptose-1,7-bisphosphate 7-phosphatase	2
**19**.	*gntK*		Thermoresistant gluconokinase	2
**20**.	*hldD*	*yqiF // rfaE // gmhC // waaE*	ADP-L-glycero-D-manno-heptose-6-epimerase	2
**21**.	*holD*		DNA polymerase III subunit psi	2
**22**.	*metR*		HTH-type transcriptional regulator MetR	3
**23**.	*narL*	*frdR // narR*	Nitrate/nitrite response regulator protein NarL	2
**24**.	*nuoN*		NADH-quinone oxidoreductase subunit N	2
**25**.	*oxyR*	*momR // mor*	Hydrogen peroxide-inducible genes activator	3
**26**.	*pfkA*		ATP-dependent 6-phosphofructokinase isozyme 1	2
**27**.	*pgi*		Glucose-6-phosphate isomerase	2
**28**.	*pgm*	*gpmA //blu*	2,3-Bisphosphoglycerate-dependent phosphoglycerate mutase	3
**29**.	*phoQ*		Sensor protein PhoQ	2
**30**.	*ppc*	*asp // glu*	Coenzyme A biosynthesis bifunctional protein CoaBC	2
**31**.	*priA*	*srgA*	Primosomal protein N′	3
**32**.	*purA*	*adeK*	Adenylosuccinate synthetase	3
**33**.	*pyrE*		Orotate phosphoribosyltransferase	2
**34**.	*rbfA*	*sdr-43 // yhbB*	30S ribosome-binding factor	2
**35**.	*rfaC*	*rfa-2 // waaC // yibC*	Lipopolysaccharide heptosyltransferase 1	3
**36**.	*rfaE*	*hldE // yqiF // gmhC // waaE*	Bifunctional protein HldE	2
**37**.	*rfaG*	*waaG*	Lipopolysaccharide core biosynthesis protein RfaG	2
**38**.	*rnt*		Ribonuclease T	3
**39**.	*rpe*	*dod // yhfD*	Ribulose-phosphate 3-epimerase	2
**40**.	*sstT*	*ygjU*	Serine/threonine transporter SstT	2
**41**.	*surA rfaD*	*waaD // hldD // htrM // nbsB*	Chaperone SurA	3
**42**.	*thyA*		Thymidylate synthase	3
**43**.	*tolA*	*cim // excC // lky // tol-2*	Tol-Pal system protein TolA	2
**44**.	*tpiA*	*Tpi*	Triosephosphate isomerase	2
**45**.	*truA*	*asuC // hisT // leuK*	tRNA pseudouridine synthase A	3
**46**.	*ubiC*		Chorismate pyruvate-lyase	2
**47**.	*umuD*		Protein UmuD	2
**48**.	*ybaP*		TraB family protein YbaP	2
**49**.	*yccM*		Putative electron transport protein YccM	2
**50**.	*ydcE*	*pptA*	Tautomerase PptA	2
**51**.	*ydcx*	*ortT*	Orphan toxin OrtT	2
**52**.	*ydeU*	*orfT // ydeK // ydeU // b1509 // b1510 // ECK1502*	AIDA-I family autotransporter YneO	2
**53**.	*yegS*		Lipid kinase YegS	2
**54**.	*yfbB*	*menH*	2-Succinyl-6-hydroxy-2,4-cyclohexadiene-1-carboxylate synthase	2
**55**.	*yfeH*		Putative symporter YfeH	2
**56**.	*yfgL*	*bamB*	Outer membrane protein assembly factor BamB	2
**57**.	*ygfZ*	*yzzW*	tRNA-modifying protein YgfZ	2
**58**.	*yheM*	*tusC*	Protein TusC	2
**59**.	*yhhH*		PF15631 family protein YhhH	2
**60**.	*yigL*		Pyridoxal phosphate phosphatase YigL	2
**61**.	*yihE*	*orfA // srkA*	Stress response kinase A	2
**62**.	*yjeK*	*epmB*	L-Lysine 2,3-aminomutase	2
**63**.	*yncA*	*mnaT*	L-Amino acid N-acyltransferase MnaT	2
**64**.	*ypjD*		Inner membrane protein YpjD	2

^*a*^ The number of points indicates the number of biological repetitions in which no growth was observed.

### Determination of bactericidal aBL activity against mutants with increased sensitivity

**Fig 4:**
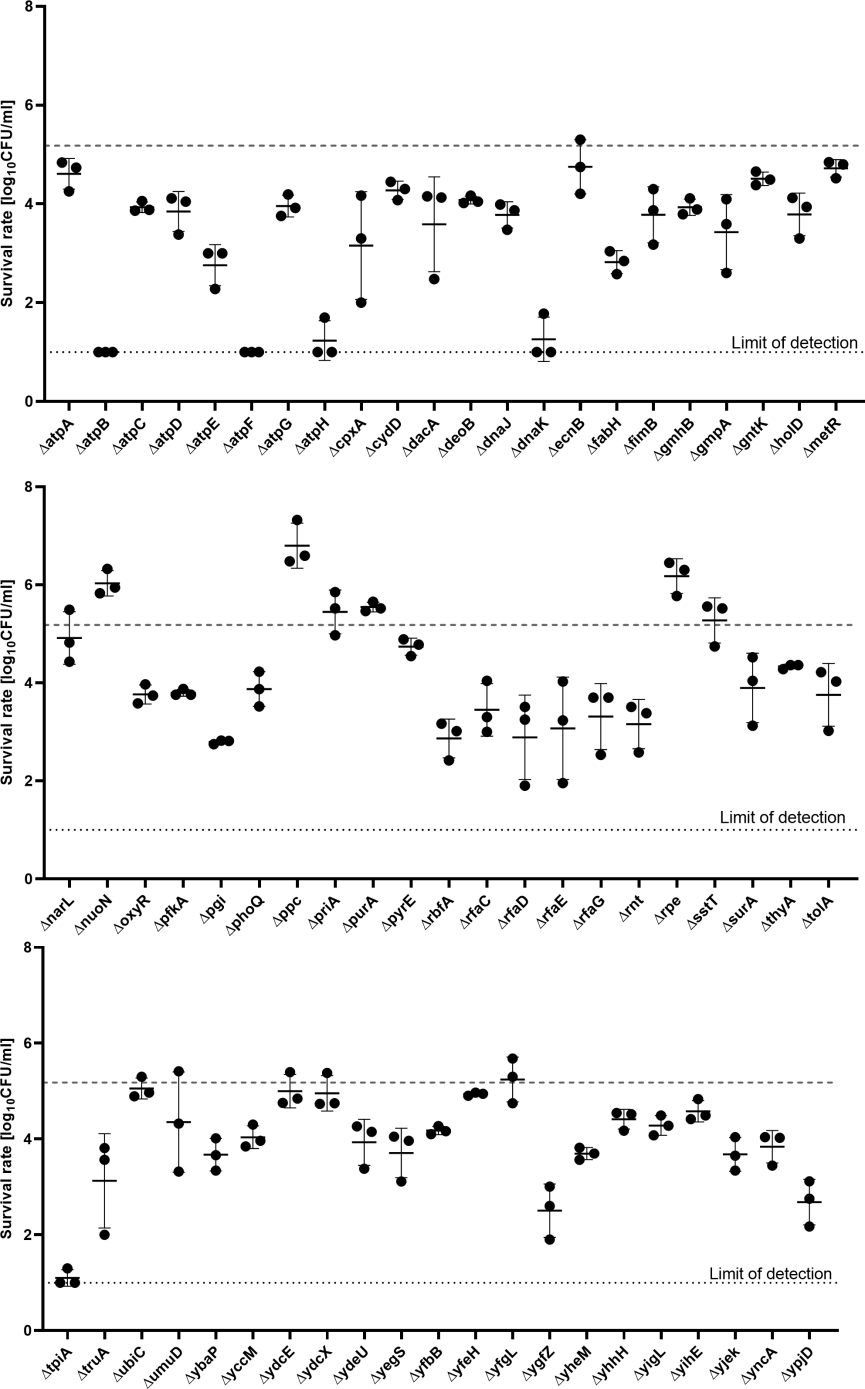
Comparison of the survival rates of the single-gene mutants irradiated with 43.2 J/cm^2^ of aBL and the BW25113 strain (indicated with dashed lines). The detection limit was 10 CFU/mL. Overnight cultures of the wild-type and mutant strains were diluted to 0.5 McF in the medium and then irradiated with 43.2 J/cm^2^ of aBL. The CFU/mL was estimated with serial dilutions of 10 µL aliquots of the irradiated samples and plated on LB agar. The plots present the reduction of log_10_ units of the CFU/mL. The experiment was performed in three biological repetitions. The value is the mean of the three separate experiments, with the bars as the ±SD of the mean.

**Fig 5:**
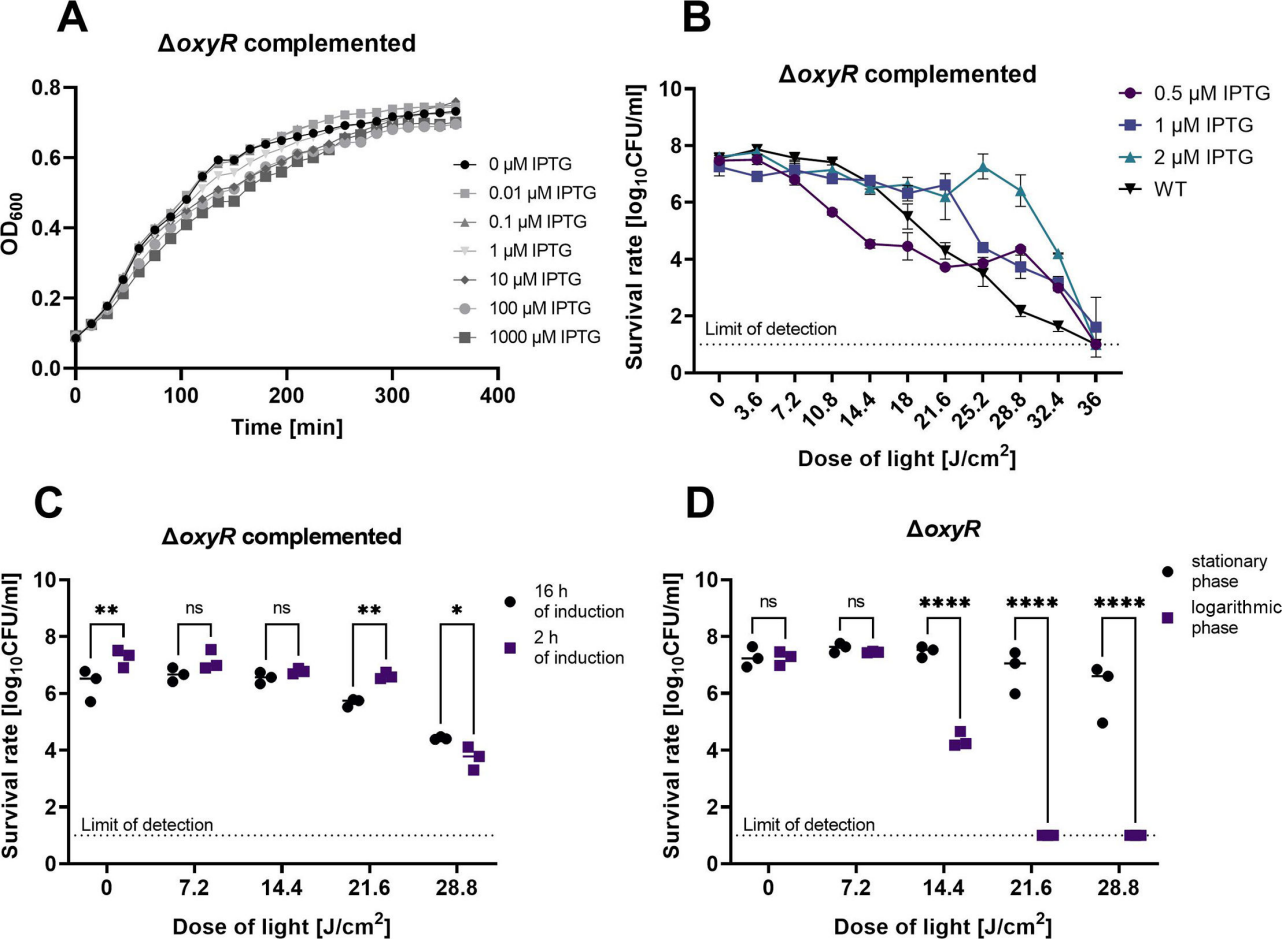
Evaluation of the *E. coli* BW25113 Δ*oxyR* deletion mutant and the complemented strain response to aBL. (A) Growth curve of the complemented Δ*oxyR* strain cultured with different IPTG concentrations. Overnight cultures of the strain were diluted at the vol/vol ratio of 1:20 and supplemented with IPTG to obtain the final concentrations of 0–1,000 µM. The growth was monitored for 6 h. The OD_600_ was measured every 15 min. (B) Comparison of the complemented Δ*oxyR* strain response to aBL depending on the IPTG concentration. Overnight cultures of the complemented strain were diluted at the vol/vol ratio of 1:100 in the fresh LB medium supplemented with specified antibiotics. The IPTG solution was added to cultures to obtain total concentrations of 2, 1, and 0.5 µM. After 2 h of incubation (37°C, 150 rpm), the cultures were diluted to 0.5 McF and treated with 0–36 J/cm^2^ of light doses. (C) Comparison of the complemented Δ*oxyR* strain depending on the growth phase and the induction time. The 16 and 2 h cultures were induced with 1 µM IPTG and treated with 0–28.8 J/cm^2^ light doses. (D) Comparison of the aBL sensitivity profiles of the Δ*oxyR* mutant depending on the growth phase. Overnight cultures (16 h, stationary phase) and log-phase cultures (2 h, exponential phase) were irradiated with 0–28.8 J/cm^2^ light doses. All the experiments were performed in three biological repetitions. The detection limit was 10 CFU/mL. The value is the mean of the three separate experiments, and the bars give the ±SD of the mean. Significance at the respective *P* values is marked with asterisks (ns *P* > 0.05; *P < 0.05; **P < 0.01; ***P < 0.001; ****P ≤ 0.0001).

To check differences in the aBL sensitivity of the hypersensitive mutants, the bactericidal aBL activity was evaluated against all the identified mutants with increased sensitivity. The survival rate of each mutant was determined and compared with the wild-type BW25113 strain survival rate after aBL treatment. Most of the strains expressed higher sensitivity to aBL than to the BW25113 strain, with a few exceptions [i.e., ∆*narL*, ∆*priA*, *∆ubiC*, and *∆yfgL* (revealing parental-like response to aBL) and *∆nuoN*, *∆ppc*, *∆purA*, *∆rpe*, and *∆sst* (expressing lower aBL sensitivity compared to the parental strain; Fig. 4)]. Lower doses of irradiation were presented in Fig. S2. Selected single-gene deletion mutants were demonstrated as significantly more susceptible to aBL in the exponential growth phase (2 h) than in the stationary growth phase (16 h) (Fig. 5D; Fig. S3).

### Determination of the appropriate IPTG concentration for the complementation experiments

Complementation was performed with ASKA plasmids (NIG, Shizuoka, Japan) for 12 selected mutant strains (i.e., ∆*rbfA*, ∆*oxyR*, ∆*dnaK*, ∆*dnaJ*, ∆*purA*, ∆*fimB*, ∆*yihE*, ∆*ydcX*, ∆*umuD,* ∆*pgi,* ∆*cpxA*, and ∆*deoB*). These knockout mutants were chosen as they lack genes for which involvement in aBL response in *E. coli* could be rationalized (i.e., DNA repair, oxidative stress response, protein stress, or others). Complementation was verified using PCR gene detection.

Next, the optimal IPTG concentration was chosen for gene expression in the complemented *oxyR* deletion strain (with IPTG-inducible promoter). The impact of different IPTG concentrations on the complemented strain was verified (Fig. 5A; Fig. S4). The results showed that the range of IPTG lowest concentrations does not significantly affect the growth of the complemented strains, as well as in the case of the WT strain, WT harboring the empty vector (pCA24N) and one selected mutant (∆*oxyR*). The highest IPTG concentrations like 100 and 1,000 µM affect the growth of *cpxA, umuD,* and *yihE* single-gene mutants in comparison to the growth without IPTG.

In the next step, based on the *oxyR* results, three IPTG concentrations were chosen: 0.5, 1, and 2 µM, and the aBL sensitivity of the 12 selected mutants was investigated. The bacterial cultures supplemented with 1 and 2 µM of IPTG responded to the aBL treatment in a manner similar to that of 21.6 J/cm^2^ of light, while higher irradiation doses caused significantly lower sensitivity of the complemented strain induced with 2 µM of IPTG. The complemented strain, induced with 0.5 µM of IPTG, was significantly more sensitive to aBL than the parental strain (Fig. 5B); thus, 1 µM of IPTG was chosen as the highest concentration that does not affect bacterial growth and results in an aBL response most similar to that of the WT strain.

Next, the aBL sensitivity of the complemented strain (induced with 1 µM IPTG) at different times of induction and growth phases was analyzed. The aBL susceptibility of the complemented strain with induced gene expression depended more strongly on the IPTG level than on the growth phase (Fig. 5C). Different phenomena occurred during native gene expression. The ∆*oxyR* aBL sensitivity depended on the growth phase. Logarithmic cultures are more sensitive to aBL than stationary cultures (Fig. 5D). In the next step, cultures in the exponential growth phase were chosen to show differences in aBL response in the phase of growth where bacterial metabolism is the most active.

### Restoration of the aBL sensitivity to at least wild-type level via complementation of the deleted genes

**Fig 6:**
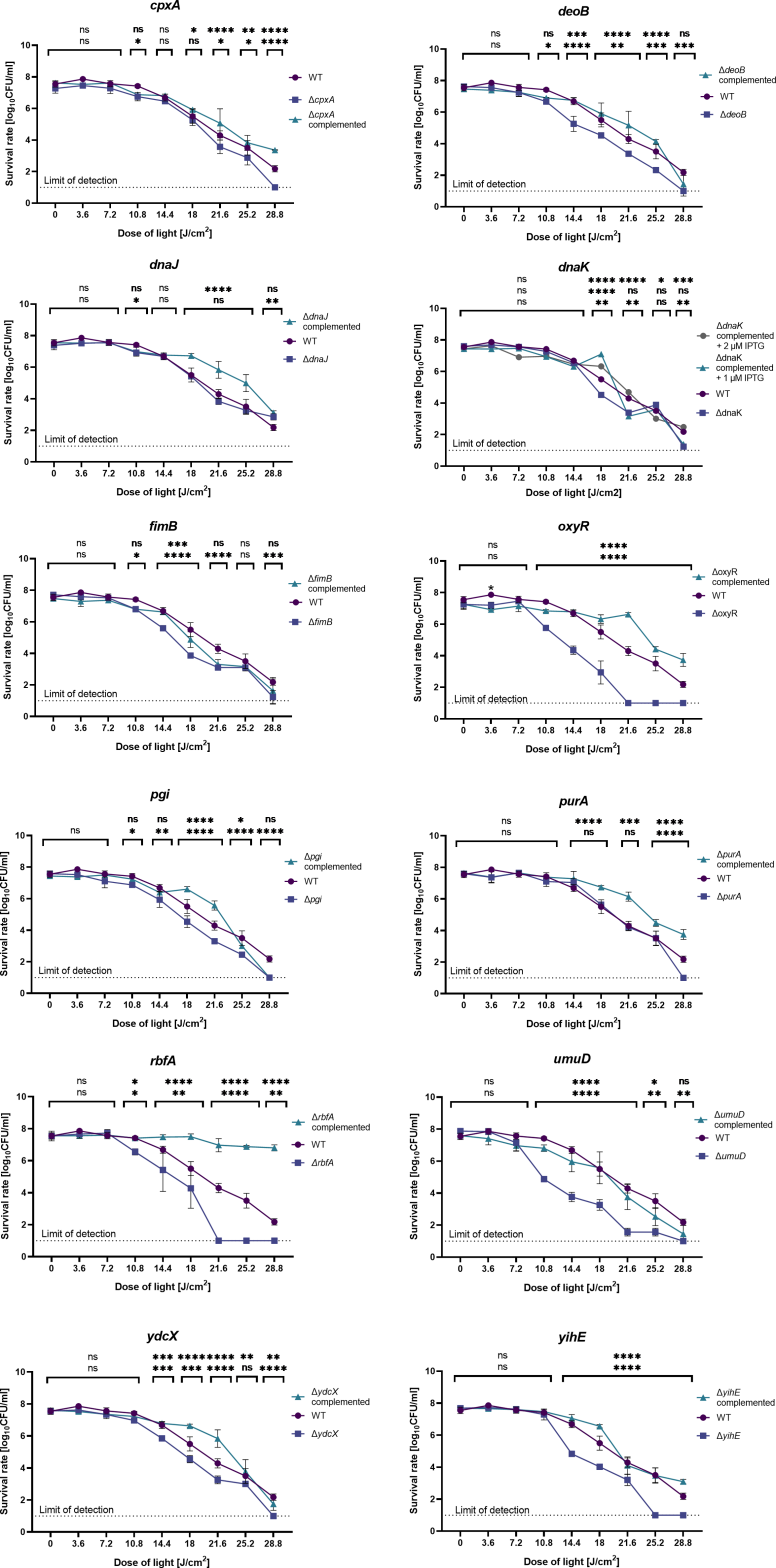
Response of the *E. coli* BW25113 (WT), single-gene deletion mutants, and complemented mutant strains to aBL. Overnight cultures of all the strains were diluted at the vol/vol ratio of 1:100 in the fresh LB medium supplemented with appropriate antibiotics. The IPTG solution was added to the complemented strain cultures to obtain a total concentration of 1 µM. After 2 h of incubation (37°C, 150 rpm), the cultures were diluted to 0.5 McF and treated with 0–28.8 J/cm^2^ aBL doses. The experiment was performed thrice. The detection limit was 10 CFU/mL. The value is the mean of the three experiments; bars represent the ±SD of the mean. The significance at the respective *P*-values is marked with asterisks (ns *P* > 0.05; *P < 0.05; **P < 0.01; ***P < 0.001). The statistical significance was tested with the deletion strain as the reference. In all the cases (except for that of the *dnaK* mutant), the upper row of asterisks refers to the comparison of the deletion mutant and the complemented strain, and the lower row of asterisks refers to the comparison of the WT and the deletion mutant strain. In the case of the *dnaK* mutant, the upmost row of asterisks refers to the comparison of the deletion mutant and the complemented strain induced with 2 µM of IPTG, and the second row from the top refers to the comparison of the deletion mutant and the complemented strain induced with 1-µM IPTG.

The aBL responses of the selected single-gene mutants, the wild-type strain, and the complemented strains were compared. First, in most cases, the complementation of the mutation restored the wild-type phenotype or made the complemented strain even more tolerant to aBL treatment (Fig. 6). The most significant differences in the aBL sensitivity of the single-gene deletion mutants and the wild-type strains were noticed for *oxyR*, *ydcX*, *yihE*, *rbfA*, *fimB*, *umuD*, and *deoB*. Though all of the 12 tested strains exhibited the phenotype with increased sensitivity to aBL through screening, the strains that lacked *purA*, *dnaK*, *cpxA*, *dnaJ*, or *pgi* responded similarly to aBL as the parental strain during further quantitative analysis. In the case of the mutants that lacked *purA*, *cpxA*, *dnaJ*, or *pgi*, the complementation of the deleted genes made the strains significantly less sensitive to aBL treatment than the uncomplemented mutant. In the case of mutants lacking the *fimB* gene, complementation only partially restored the sensitivity of WT. Since the aBL response of the mutant that lacked *dnaK* was comparable to the aBL response of its complemented mutants, it can be assumed that IPTG concentrations were insufficient or expression failed for other reasons. Thus, in the case of *dnaK*, the analysis was also performed with 2-µM IPTG. Indeed, the increased IPTG concentration resulted in decreased aBL sensitivity of the complemented ∆*dnaK* mutant, indicating that this gene might also be involved in protection against aBL as overproduction gives full protection against the tested aBL doses.

## DISCUSSION

The efficiency of aBL is considered the result of its multitarget mode of action. Nevertheless, it must be admitted that the entire mechanism of aBL action is not yet completely understood. The most widely accepted hypothesis concerning aBL treatment indicates that the key role in aBL bactericidal activity is played by the endogenous photoreactive compounds that naturally occur in bacterial cells (i.e., coproporphyrin, uroporphyrin, and protoporphyrin). These endogenous photosensitizing compounds absorb the Sored-band light of appropriate wavelengths between 405 and 420 nm, which leads to their excitation and finally to the production of reactive oxygen species, which include singlet oxygen, hydroxyl radicals, peroxides, and superoxides (26). ROS, a toxic factor, plays a crucial role in exerting the cytotoxic effects of aBL on multiple cellular structures via protein oxidation, enzyme inactivation, DNA damage, and alterations in the lipid profiles and transmembrane potential (ion leakage), which results in microbial cell death (27 – 29).

It should be mentioned that resistance to aBL has not been described yet. Due to the multitarget mode of action of aBL, it is considered a low-risk treatment to develop bacterial resistance (30). Indeed, in our previous studies, we included data that were very supportive of this hypothesis by providing proof that even using an adequate and approved experimental resistance assessment methodology did not result in microbial resistance development to aBL treatment, though multiple aBL exposures might lead to the development of aBL-tolerant phenotypes (19, 21). Also, Luo et al. (31) revealed that after multiple sublethal aBL exposures, bacterial adaptation and changed aBL susceptibility occurred in *Staphylococcus aureus* (31). It is well known that gram-negative bacteria, represented by *E. coli*, are capable of adapting to many chemicals and physical and environmental stressors [i.e., antibiotics (32), organic solvents (33, 34), heavy metals (35, 36), acids (37, 38), UV radiation (39), and ionizing radiation (40)]. Moreover, it was also noticed that this adaptation process involved another unfavorable phenomenon, such as cross-tolerance or cross-protection. Ramteke (41) demonstrated that 90% of 448 coliform isolates showed resistance to one or more antibiotics but demonstrated tolerance to multiple metals (41). Rowe and Kirk (42) investigated the phenomenon of cross-protection in *E. coli* O157:H7 and reported increased salt or heat tolerance when the bacteria were prestressed with acid, which indicated that this could affect food processing (42). For this reason, it is of high importance to analyze the possible development of microbial tolerance and/or resistance to any new and alternative antimicrobial approach (i.e., visible light-based therapies).

In this study, we performed screening analysis using the Keio knockout collection. This collection is an extensively studied and adequate genetically based tool for determining the effects of single-gene deletions under different stress conditions and performing genome-wide analysis (22). It has already been used for research, including on biofilm formation (43), swarming (44), growth in human blood (45), antibiotic hypersensitivity (46, 47), antibiotic resistance (48), non-thermal atmospheric pressure plasma hypersensitivity (24), hydroxyurea sensitivity (49), control of bacterial conjugation of antibiotic resistance (50), cysteine tolerance (51), colicin cytotoxicity (52), oxidizing agent resistance (53), copper stress (54), tolerance to chelants (55), susceptibility to microcin PDI (56), and glycogen metabolism (57). For instance, Mohiuddin et al. (58) determined genes critical for bacterial persistence and identified 55 genes of ofloxacin persistence and 50 genes related to ampicillin resistance (58). Krewing et al. (24) found 87 mutants that exhibited increased plasma sensitivity (24). Chen et al. (53) performed genome-wide analysis to identify genes involved in oxidizing agent resistance and identified 114 candidate genes related to HOCl resistance and 217 genes associated with resistance to H_2_O_2_. Of all the identified genes, 63 (in the case of HOCl) and 105 (in the case of H_2_O_2_) have not yet been associated with oxidative stress response (53). Those results proved that genome-wide analysis enables the exploration of unknown mechanisms of bacterial response to various factors and supplies novel data for the development of new therapeutic approaches. Referring to Nakayashiki and Mori (49), the genome-wide screening may reveal the emergence of unexpected clones, indicating that scientists do not yet understand all aspects of gene functions or interactions between genes in bacterial intracellular networks (49).

**Fig 7:**
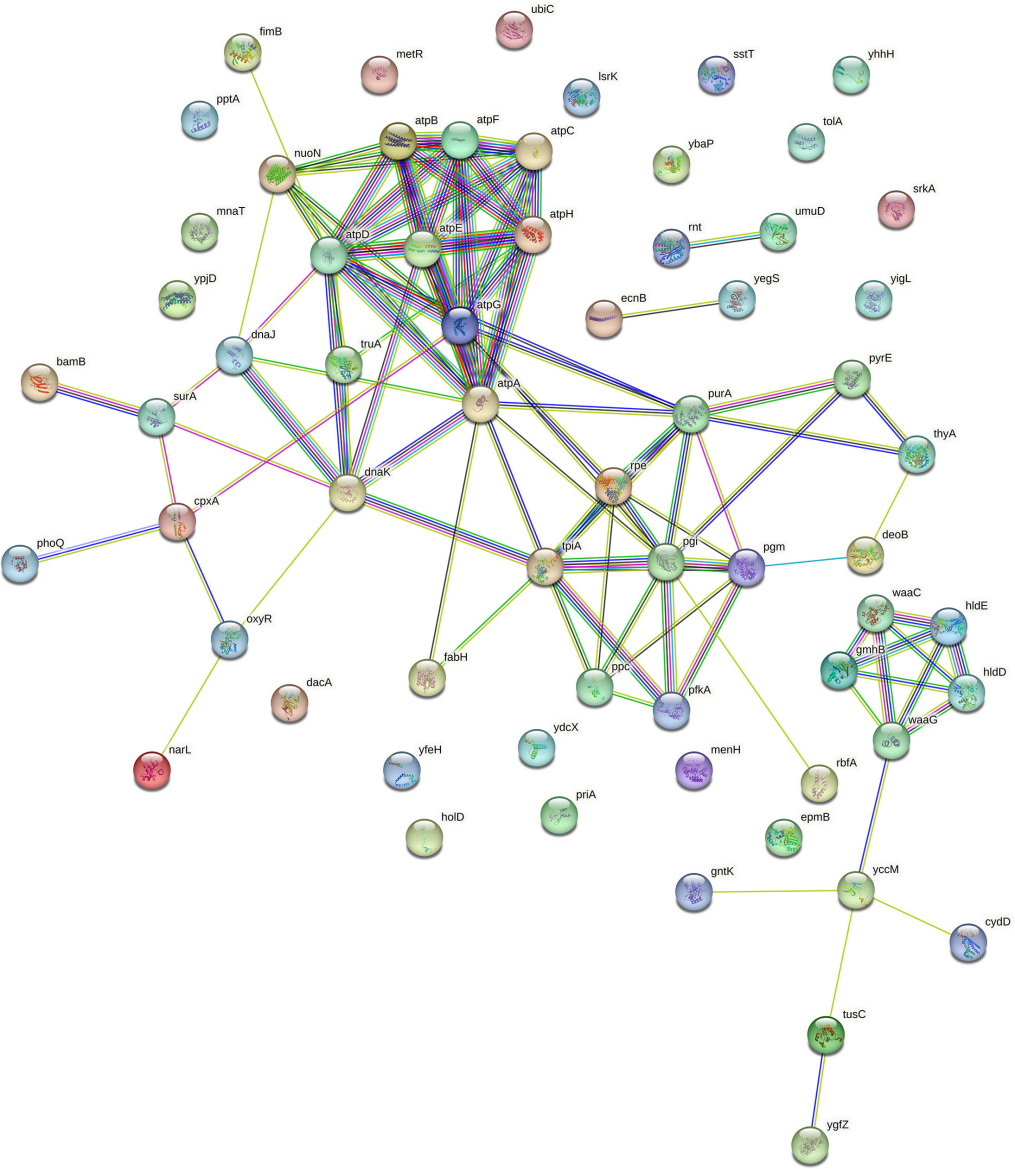
Protein–protein functional interaction networks. Protein–protein functional interaction networks of the proteins encoded by 64 aBL-protective genes. The analysis was performed with the STRING database with a medium confidence score of 0.4. The colors of the lines denote the following: light blue, interactions known from curated databases; pink, interactions experimentally determined; bright green, predicted reaction (gene neighborhood); red, gene fusions; dark blue, gene co-occurrence; green, textmining; black, co-expression; and blue, protein homology.

In this study, we screened almost 4,000 single-gene mutants and demonstrated 64 aBL-protective genes that could potentially be involved in the development of tolerance or resistance of *E. coli*, for example, due to genetic alterations that would lead to their overexpression. The performed analysis revealed that these genes contribute to a wide range of biological processes, mainly biosynthesis, metabolism, regulation, stress response, DNA repair, and others. The STRING database analysis revealed interactions and functional associations between the proteins encoded by the identified genes (Fig. 7).

During the screening, we identified 64 knockout mutants with extended sensitivity to aBL. After further analysis, the majority of the mutants exhibited hypersensitivity, some of them (e.g., *ΔpyrE* and *ΔyfeH*) exhibited a moderate increase in sensitivity, and only a few of them showed similar sensitivity to the WT strain (e.g., *ΔubiC* and *ΔyfgL*). However, we chose to include all mutants identified by screening in the further analysis, as the lack of growth in at least two out of three replicates was not due to growth defects, which was supported by appropriate controls and what is consistent with the observations of the authors of the Keio collection, that most of the mutants did not show any distinct growth defects in rich media (22). The discrepancies may also result from the difference in the density of the bacteria used in the qualitative (screening) and quantitative (profiles of the aBL sensitivity) tests.

Due to the performed analysis, we were able to identify several genes, which, if lacking, may lead to aBL increased sensitivity and can be engaged in the cellular response to this treatment. One of them may be *oxyR*. OxyR protein, termed an “oxidative stress regulator,” is responsible for the protection of bacterial cells against the toxicity caused by ROS. Furthermore, multiple studies revealed that the key role of *oxyR* is to regulate the expression of numerous genes involved in the microbial response to oxidative stress (59, 60). Though the existence of this direct link of *oxyR* to stress response has been established, no report has indicated the direct impact of *oxyR* on *E. coli* sensitivity to aBL. This study demonstrated that cells that lack the *oxyR* product are more susceptible to aBL treatment, and on the other hand, the complementation of ∆*oxyR* deletion restored the wild-type phenotype and increased strain tolerance to aBL. The results of this study supported the hypothesis that aBL generates ROS, which plays a crucial role in aBL toxicity against microbial cells (17). *oxyR* regulates the expression of numerous genes (i.e., *metR,* the deletion of which was also demonstrated to result in aBL hypersensitivity). However, the expression level of *oxyR* in the complemented strain can be much higher than that in the WT, as OxyR serves as both a transcriptional activator and a repressor of *oxyR* transcription (61).

According to the functional analysis of hypersensitive genes performed using the BioCyc database and the Omics Dashboard, 12.5% of all the identified genes (eight genes; i.e., *umuD*, *rnt*, *rbfA*, *priA*, *oxyR*, *purA*, *fimB*, and *deoB*) were involved in the cellular response to DNA damage. This might have been expected, as the bacteria increased the adaptation potential of the genes by modulating the rate of mutation (62). In addition, the aBL exerts mutagenic potential and could trigger a repair response. McGinty and Fowler observed base-pair substitution (transversions at both G:C and A:T sites) and frameshift mutations in *E. coli* induced with blue light irradiation (450 nm) (63). One of the genes investigated in this study, *umuD*, encodes UmuD, which is a part of the *E. coli* umuD’2C complex (PolV SOS), an error-prone DNA polymerase responsible for UV protection and the main factor that leads to SOS mutagenesis. It enables DNA replication across DNA lesions (64). Interestingly, in our previous studies, we observed no development of adaptation to aBL in an *umuD*-deficient mutant of *E. coli* BW25113 when it was subjected to multiple sublethal aBL treatments ((in comparison to the parental strain) (21). Next, RNase T, the product of the *rnt*, was identified as involved not only in tRNA processing but also in single-stranded DNA degradation, which may suggest its role in DNA repair. Viswanathan et al. (65) revealed that RNase T may serve as a high-copy suppressor of UV sensitivity in DNA exonucleases-deficient *E. coli* mutants (65). Another gene, whose deficiency resulted in the highest aBL sensitivity, is *rbfA*. This gene encodes the 30S ribosome binding factor responsible for ribosomal maturation and/or the initiation of translation and is suggested to be a cold-shock protein. Jones and Inouye (66) showed that the absence of *rbfA* triggers the cold‐shock response; whereas in the case of RbfA overproduction, it resulted in increased total protein synthesis and faster growth adaptation to the lower temperature (66). Moreover, Rooney et al. (67) highlighted the role of RbfA in DNA damage response during their investigation of DNA repair after the alkylation process (67).

The next identified gene (*priA*) encodes a polypeptide PriA, which is required for immediate restarting of DNA synthesis after UV irradiation (68). It has also been demonstrated, in the case of *Bacillus subtilis*, that PriA is a crucial factor for microbial survival after severe DNA damage induced by numerous antibiotics (69) and is an essential factor for the survival and persistence of *Helicobacter pylori* in mouse stomach mucosa (70). Another gene detected in this study was *purA*, which encodes adenylosuccinate synthetase that is responsible for catalyzing *de novo* synthesis of AMP. Sun et al. demonstrated that the DNA damage induced by acidic conditions was significantly higher in *purA-*deficient mutants than in wild-type *E. coli*. These results clearly indicate that microbial survival in extreme environmental conditions involves metabolic processes that require ATP, i.e., an ATP-dependent DNA repair system (71). This is in line with our results, indicating that deficiency in one of the seven genes involved in ATP processing pathways (i.e., *atpA*, *atpB*, *atpC*, *atpE*, *atpF*, *atpG*, and *atpH*) results in aBL hypersensitivity. All the mentioned genes encode ATP synthase (F0F1 synthase) complex subunits required for ATP biosynthesis.

The analysis performed with the use of BioCyc database and the Omics Dashboard indicated that almost half (48%) of hypersensitive gene products are localized at a cell exterior where plasma membrane proteins (36% of all genes), lipopolysaccharide metabolism proteins (7.8%), and outer membrane proteins (4.7%) can be partly found. It may support the generally accepted thesis that bacterial envelopes serve as primary targets of blue light treatment, and these microbial structures are a crucial part of a bacterial first line of defense against the damaging effects of aBL (18, 72, 73).

An example of an inner membrane protein encoded by one of the identified genes was the orphan toxin gene (*ortT*). OrtT is a protein toxin activated under conditions that induce a stringent response. OrtT reduces cell growth and metabolism during nutritional or antimicrobially caused stress (e.g., trimethoprim and sulfamethoxazole), leads to cell membrane damage, and reduces the intracellular ATP level (74). Next, *ecnB*, which is a part of an antidote/toxin system (entericidin), plays a role in starvation adaptation by supporting further cell growth at the expense of dying subpopulations and is involved in programmed bacterial cell death (75). Segura et al. (76) revealed that *ecnB* could be one of the factors involved in the persistence of a Shiga toxin-producing *E. coli* O157:H7 strain in bovine intestine content (76). Another gene that encodes inner membrane protein is *tolA*. TolA interacts with the *E. coli* porins (e.g., OmpF) and is essential for the functionality and stability of the *E. coli* outer membrane (77). *E. coli tolA* contains a highly variable tandem repeat region (78, 79), the size of which was demonstrated to contribute to the fitness of *E. coli* under specific stress conditions, influencing its tolerance (79). Our previous study also supported the assumption of the significant role of *tolA* in aBL response, as the *tolA*-deficient *E. coli* BW25113 mutant was significantly more sensitive to aBL than the wild-type strain and did not develop a significant aBL tolerance, unlike the wild-type strain (21).

Finally, in this study, two heat shock proteins (DnaJ and DnaK) were also demonstrated to play an aBL-protective role. This corresponds with another study by Kim et al. (80) concerning oxidative stress, reporting that DnaK/DnaJ chaperone protects *Salmonella* against the cytotoxic effects resulting from ROS activity (protection against protein carbonylation) (80).

One of the key point of our study was to determine the proper irradiation conditions that were sublethal (MDK_99_) for the wild-type *E. coli* BW25113 strain but could be lethal (≥MDK_99.9_) for aBL-sensitive mutants. The dose–exposure time relationship in the context of phototherapy is an important issue. We have knowledge from the previous studies of our research group (81) that the irradiation power influences mortality dynamics. We have observed that the greater the irradiation power, the greater the bacterial mortality. The mortality curves reach the detection limit at various irradiation time when the irradiation power is changed, and we can conclude that the detection limit is reached faster when irradiation power is higher (and the time of exposure is shorter). Moreover, we are convinced that the exposure time of irradiation could influence the gene expression and has impact on activating protective and repair mechanisms in the bacterial cell. For example, the biological half-life of SOS repair is 20–30 min (according to the decay of error-prone repair activity) (82). Taking into account the above and the time needed for one generation of bacteria, the exposure time (defined as “sublethal dose,” at the maximum power of the light source) of 30 min (used for screening, i.e., qualitative analysis, where the inoculum was higher) or 20 min (used for quantitative analysis, where the inoculum was lower) seemed optimal.

Foodborne diseases are mainly associated with ingesting contaminated water and food. Gram-negative bacteria are predominant in food-processing environments (2). Antimicrobial resistance is a challenge that is not restricted to clinics but also the food industry and wastewater. Wastewater, along with wastewater treatment plants, is not only reservoirs but also “hotspots” for horizontal gene transfer (83). Antimicrobial blue light is a promising strategy for decontaminating food products (84 – 86) and surfaces (84, 87, 88). aBL is still seen as demonstrating an advantage over antibiotic treatment, which involves low-risk resistance development, due to its multitarget mode of action. Indeed, the development of resistance to aBL has never been observed. However, reports on aBL tolerance cannot be unequivocally ruled out; over time, the phenomenon of adaptation to aBL will be observed. We managed to indicate 64 aBL-protective genes that could be the potential candidates for the development of tolerance or resistance of *E. coli* to this visible light treatment. This study can contribute to a better understanding of bacterial response to aBL and makes this treatment an attractive alternative bactericidal approach not only in clinical use but also in environmental application.

## SUPPLEMENTAL MATERIAL

Supplemental material spectrum.02490-23-s0001.docx Supplemental figures and table.

Graphical abstract spectrum.02490-23-s0002.tif Graphical abstract.

ASM does not own the copyrights to Supplemental Material that may be linked to, or accessed through, an article. The authors have granted ASM a non-exclusive, world-wide license to publish the Supplemental Material files. Please contact the corresponding author directly for reuse.
