# Supplementary material for: Can antimicrobial blue light contribute to resistance development? Genome-wide analysis revealed aBL-protective genes in Escherichia coli
Source: Microbiol Spectr. 2023 Dec 8;12(1):e02490-23. doi: 10.1128/spectrum.02490-23 (PMC10782963; doi:10.1128/spectrum.02490-23)
Supplement: Supplemental material — Supplemental figures and table. [file spectrum.02490-23-s0001.docx]

Supplementary data

**Supplementary Table. 1. Hyperensitive genes and their functions**

| Function | | | | Name of gene |
| --- | --- | --- | --- | --- |
| Biosynthesis | Nucleotide and Nucleoside biosynthesis | Purine biosynthesis | | *purA* |
|  |  | Pyrimidine biosynthesis | | *thyA*  *pyrE* |
|  | Fatty acids and lipid biosynthesis | Fatty Acid Biosynthesis Initiation | | *fabH* |
|  |  | Lipid A- core biosynthesis | | *waaC*  *waaG* |
|  | Cofactor, Carrier, Vitamin synthesis | Electron Carrier Biosynthesis | | *menH*  *ubiC* |
|  |  | Folate Biosynthesis | | *thyA* |
|  | Carbohydrate synthesis | Gluconeogenesis | | *tpiA*  *pgi* |
|  |  | Glycan Biosynthesis | | *pgm*  *waaC*  *waaG* |
|  |  | Glycogen and Starch Biosynthesis | | *pgm* |
|  |  | Sugar nucleotde Biosynthesis | ADP-sugar biosynthesis | *gmhB*  *hldE*  *rfaD* |
|  |  |  | dTSP-sugar biosynthesis | *pgm* |
|  |  |  | GDP-sugar biosynthesis | *pgi* |
|  |  |  | UDP-sugar biosynthesis | *pgm*  *pgi* |
|  | Other | 4-hydroxybenzoate | | *ubiC* |
| Degradation | Nucleotide and Nucleoside degradation | Purines and pyrimidines | | *deoB* |
|  | Carbohydrates and Carboxylates degradation | Carbohydrates | | *pgm*  *deoB*  *yigL* |
|  |  | Carboxylates degradation | | *gntK* |
| Energy | Glycolisis | | | *pfkA*  *tpiA*  *pgi* |
|  | Pentose Phosphate Pathway  (PPP) | | | *rpe* |
|  | Aerobic and anaerobic respiration | | | *nuoN* |
|  | ATP biosynthesis | | | *atpG*  *atpF*  *atpA*  *atpH*  *atpC*  *atpE*  *atpB*  *atpD* |
|  | Other | sedoheptulose bisphosphate bypass | | *pfkA* |
|  | DNA Metabolism | Replication | | *dnaK*  *dnaJ*  *rnt*  *priA*  *holD* |
|  |  | Recombination | | *priA*  *fimB* |
|  |  | Repair | | *umuD*  *rnt*  *priA* |
|  |  | Integration | | *fimB* |
|  | RNA Metabolism | RNA Processing | | *rnt*  *rbfA* |
|  |  | tRNA Processing | | *rnt*  *truA*  *ygfZ*  *tusC* |
|  |  | Regulation of RNA Methabolic Process | | *dnaK*  *dnaJ*  *umuD*  *narL*  *metR*  *oxyR* |
|  |  | Other Proteins involved in RNA Metabolism | | *priA* |
|  | Protein Metabolism | Proteolysis | | *dacA*  *umuD* |
|  |  | Regulation | | *thyA* |
|  |  | Other Proteins involved in Protein Metabolism | | *phoQ*  *epmB*  *cpxA* |
|  | Protein Folding | | | *surA*  *dnaJ*  *dnaK* |
| Cellular Processes | Cell Cycle and Division Proteins | | | *dacA*  *tolA* |
|  | Proteins involved in cell death | | | *ortT* |
|  | Proteins involved in Biofilm formation | | | *cpxA* |
|  | Proteins Involved in response to virus | | | *tolA*  *dnaJ* |
|  | Proteins Involved in interaction with Host and Symbiosis | | | *tolA* |
| Cell Exterior | Transport | Transporters of Amino Acids and their Derivatives | | *cydD*  *sstT* |
|  | Cell Wall Biogenesis/Organization Proteins | | | *dacA* |
|  | Lipopolysaccharide Metabolism Proteins | | | *gmhB*  *waaG*  *waaC*  *rfaD*  *hldE* |
|  | Outer Membrane Proteins | | | *yneO*  *bamB*  *ecnB* |
|  | Plasma Membrane Proteins | | | *dnaK dacA tolA cydD yccM phoQ ortT yneO nuoN yfeH ypjD sstT yhhH waaC atpC atpG atpA atpH atpF atpE atpB cpxA ecnB* |
|  | Periplasmic Proteins | | | *surA*  *dacA* |
| Response to stimulus | Proteins Involved in Response to Starvation | | | *phoQ* |
|  | Proteins Involved in Response to Heat | | | *dnaK*  *dnaJ* |
|  | Proteins Involved in Response to Cold | | | *rbfA* |
|  | Proteins Involved in Response to DNA Damage | | | *umuD rnt rbfA priA oxyR purA fimB deoB* |
|  | Proteins Involved in Response to osmotic Stress | | | *phoQ* |
|  | Other Proteins involved in Stimulus Response | | | *tolA*  *holD*  *ecnB*  *pgi*  *cpxA*  *srkA*  *narL*  *yigL* |
| Regulation | Signaling | CpxAR Two-Component Signal Transduction System | | *cpxA* |
|  |  | NarQ Two-Component Signal Transduction System, nitrate dependent | | *narL* |
|  |  | NarX Two-Component Signal Transduction System, nitrate dependent | | *narL* |
|  |  | PhoQP Two-Component Signal Transduction System, magnesium-dependent | | *phoQ* |
|  | Sigma Factors Regulons | Regulon of RNA polymerase sigma factor RpoD | | *cydD fabH umuD narL rnt nuoN thyA rbfA rpe gntK rfaD waaC waaG atpC atpG atpA atpH atpF atpE atpB srkA cpxA pfkA tpiA oxyR pgi, ubiC purA fimB deoB* |
|  |  | Regulon of RNA polymerase sigma factor RpoH | | *dnaK*  *dnaJ*  *phoQ*  *waaC*  *rfaD* |
|  |  | Regulon of RNA polymerase sigma factor RpoE | | *surA*  *phoQ*  *bamB*  *rfaD*  *waaC* |
|  |  | Regulon of RNA polymerase sigma factor RpoS | | *cpxA*  *ecnB*  *pgi*  *pfkA*  *tpiA*  *oxyR* |
|  | Transcription factors | | | *oxyR*  *metR*  *narL* |
|  | Transcription factor regulons | Regulon of DNA-binding transcriptional dual regulator OxyR | | *metR*  *oxyR* |
|  |  | Regulon of DNA-binding transcriptional dual regulator H-NS | | *fimB* |
|  |  | Regulon of DNA-binding transcriptional dual repressor DeoR | | *deoB* |
|  |  | Regulon of DNA-binding transcriptional dual regulator Nac | | *yccM*  *ortT*  *waaG*  *yigL*  *metR*  *ppc*  *ecnB* |
|  |  | Regulon of DNA-binding transcriptional dual regulator NagC, BasR | | *fimB* |
|  |  | Regulon of DNA-binding transcriptional dual regulator SoxS | | *pgi* |
|  |  | Regulon of DNA-binding transcriptional dual regulator PhoB, PhoP | | *phoQ* |
|  |  | Regulon of DNA-binding transcriptional activator ZraR | | *rfaD* |
|  |  | Regulon of DNA-binding transcriptional dual regulator ArcA | | *cydD*  *nuoN*  *sstT*  *ubiC* |
|  |  | Regulon of DNA-binding transcriptional repressor LexA | | *umuD* |
|  |  | Regulon of DNA-binding transcriptional activator GadE | | *purA* |
|  |  | Regulon of NanR | | *fimB* |
|  |  | Regulon of DNA-binding transcriptional dual regulator HU | | *pgm* |
|  |  | Regulon of DNA-binding transcriptional dual regulator OmpR | | *sstT*  *ecnB* |
|  |  | Regulon of DNA-binding transcriptional repressor CytR | | *deoB* |
|  |  | Regulon of DNA-binding transcriptional dual regulator FNR | | *cydD*  *narL*  *ortT*  *nuoN*  *ubiC* |
|  |  | Regulon of DNA-binding transcriptional dual regulator CpxR | | *srkA*  *cpxA* |
|  |  | Regulon of MetJ-S-adenosylmethionine | | *metR* |
|  |  | Regulon of DNA-binding transcriptional dual regulator ArcA, NarL | | *cydD*  *nuoN*  *narL*  *ubiC* |
| Other Pathways | Nitrate Reduction | | | *nuoN* |
|  | Nucleic Acid Processing | | | *rnt*  *tusC* |
|  | Enzymes not in Pathways | | | *dnaK*  *dnaJ*  *surA*  *umuD*  *mnaT*  *pptA*  *yegS*  *truA*  *srkA*  *epmB* |
| Genes not present in any subsystem | | | | *ybaP* |


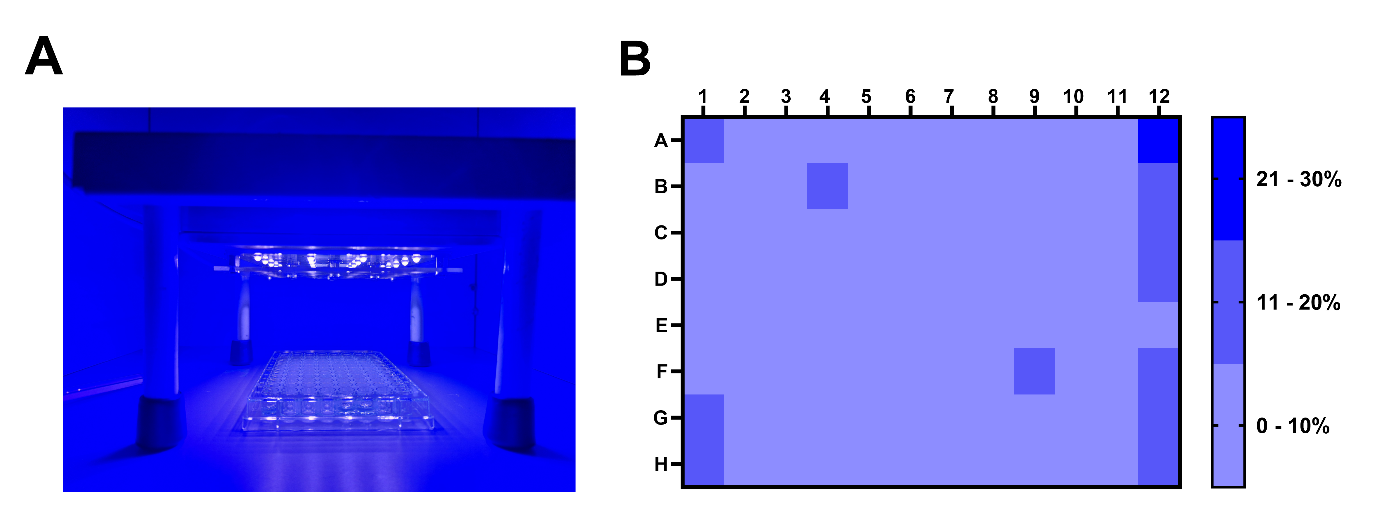


**Supplementary Figure 1. The LED light source used in research.** A. The picture of the LED light source used in the research. B. The map of irradiance distribution over the illuminated area. Percentage indicate deviations from the mean power for all irradiation areas. The LED light source was constructed to reach a homogeneous light distribution. The differences between light densities do not exceed 10% (except for a few fields, which were considered when performing the experiments). The 96-well plates from different biological replicates were placed in different orientations relative to the light source to increase power homogeneity.

**Supplementary Figure. 2 Survival rate of single gene mutants irradiated with 0-43.2 J/cm^2^ in comparison to WT (BW25113) survival rate.** The detection limit was 10 CFU/ml. Overnight cultures of wild and mutant strains were diluted to 0.5 McF in medium and then irradiated with 43.2 J/cm^2^. Colony-forming units (CFU/mL) were estimated with serial dilutions of 10 μL aliquots of irradiated samples and plated on LB agar. Plots present the reduction of log_10_ units of CFU/ml. The experiment was performed in three biological repetitions. The value is a mean of three separate experiments with bars as ± SD of the mean.

**Supplementary Figure. 3. Comparison of the aBL sensitivity profiles of the selected single gene mutants depending on the growth phase.** Overnight cultures (16 h, stationary phase) and logarithmic phase cultures (2 h, exponential phase) were irradiated with 0–28.8 J/cm^2^ light doses. All the experiments were performed in three biological repetitions. The detection limit was 10 CFU/ml. Significance at the respective p values is marked with asterisks [ns p > 0.05; ****p ≤ 0.0001].


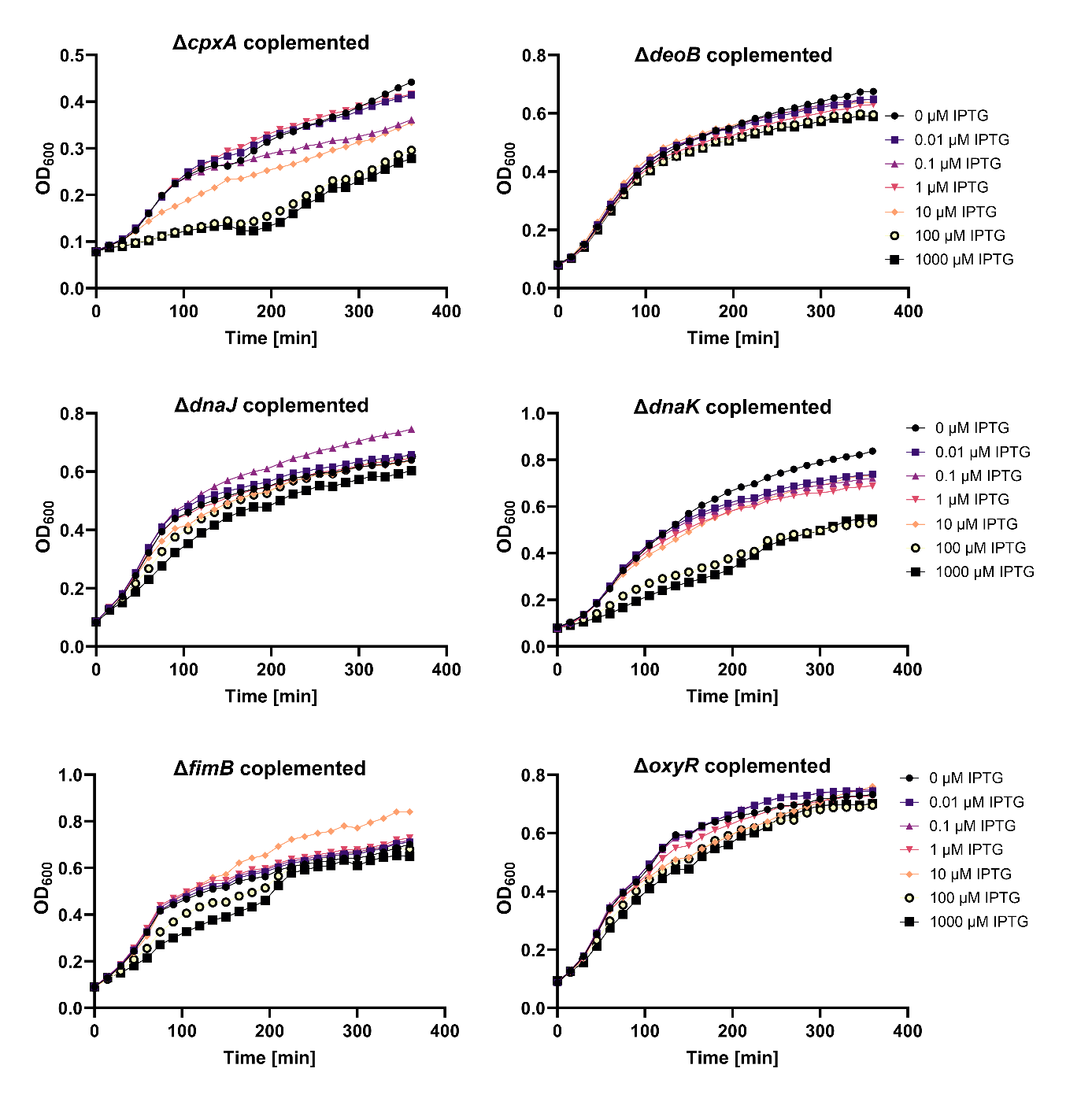


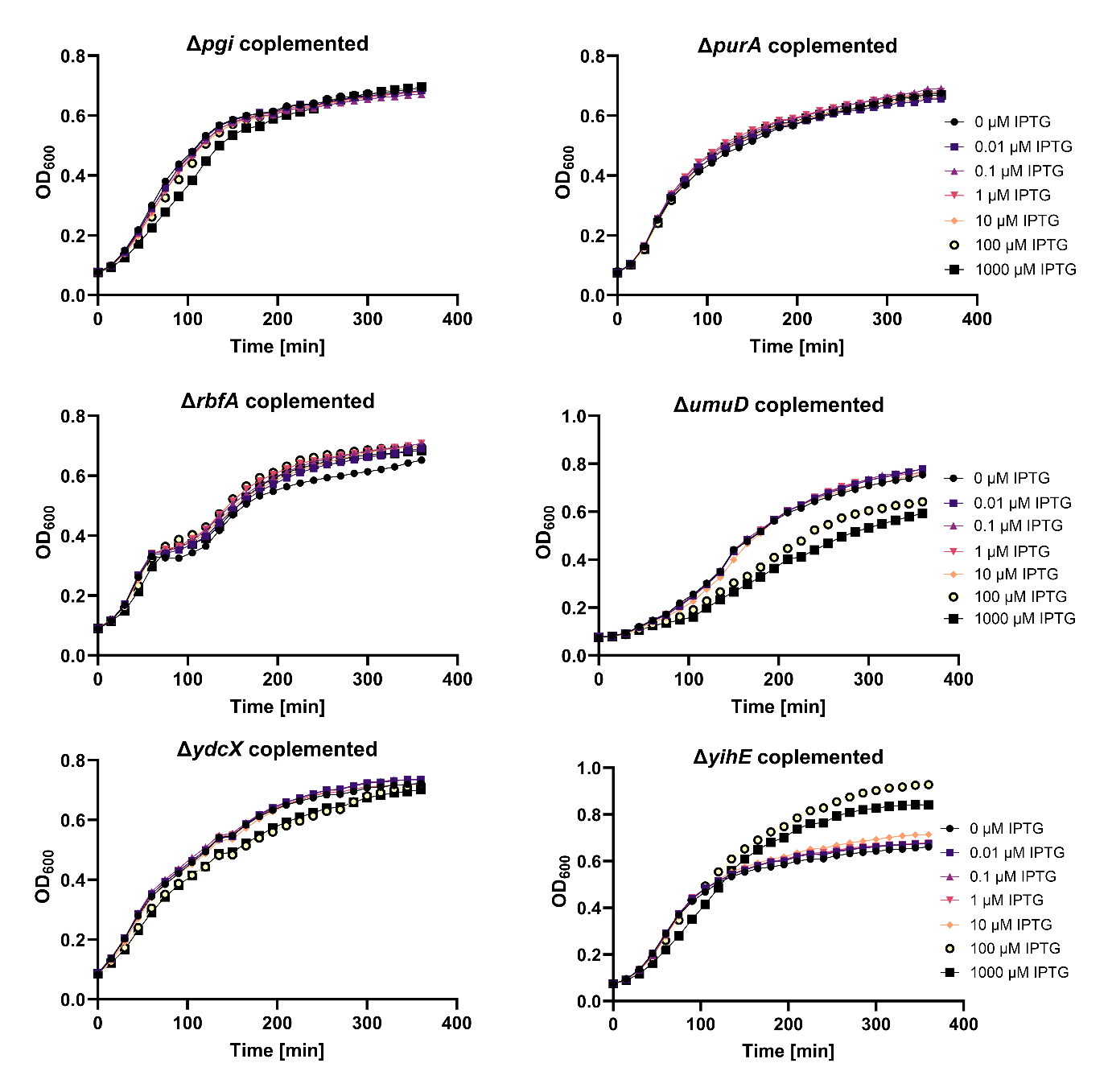


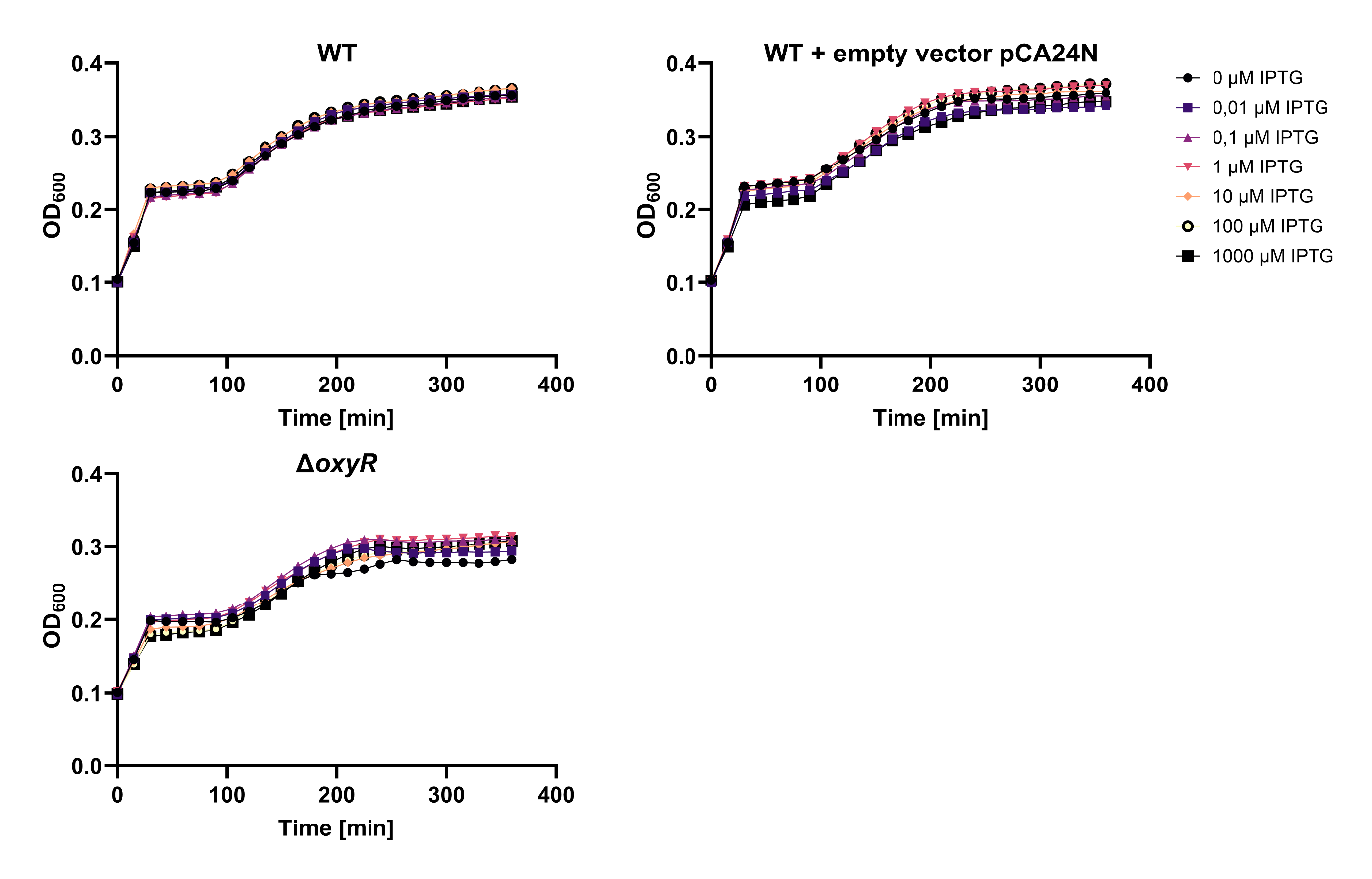


**Supplementary Figure. 4. Growth curve of the complemented strains, WT, WT harbouring the empty vector pCA24N and Δ*oxyR* mutant cultured with different IPTG concentrations.** Overnight cultures of the strain were diluted at the v/v ratio of 1:20 and supplemented with IPTG to obtain the final concentrations of 0–1000 μM. The growth was monitored for 6 h. The OD_600_ was measured every 15 min. All the experiments were performed in three biological repetitions.
