# Supplementary figures and images for: Can antimicrobial blue light contribute to resistance development? Genome-wide analysis revealed aBL-protective genes in Escherichia coli
Source: Microbiol Spectr. 2023 Dec 8;12(1):e02490-23. doi: 10.1128/spectrum.02490-23 (PMC10782963; doi:10.1128/spectrum.02490-23)

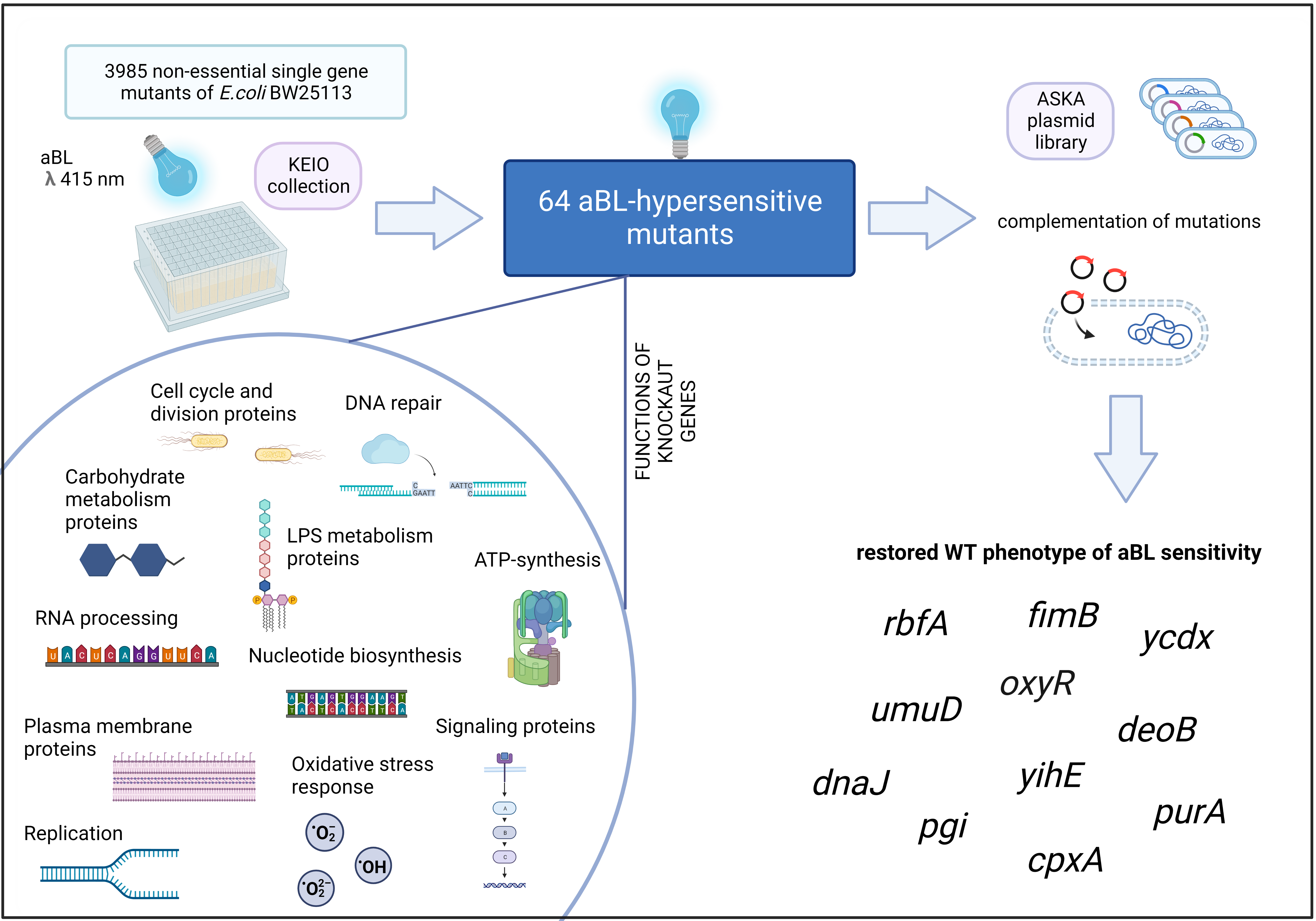

Supplement: Graphical abstract [file spectrum.02490-23-s0002.tif]
